# Long noncoding RNA DNM3OS promotes prostate stromal cells transformation via the miR-29a/29b/COL3A1 and miR-361/TGFβ1 axes

**DOI:** 10.18632/aging.102395

**Published:** 2019-11-06

**Authors:** Ruizhe Wang, Mengda Zhang, Zhenyu Ou, Wei He, Lingxiao Chen, Junjie Zhang, Yao He, Ran Xu, Shusuan Jiang, Lin Qi, Long Wang

**Affiliations:** 1 Department of Urology, Xiangya Hospital, Central South University, Changsha, Hunan 410008, China; 2 National Clinical Research Center for Geriatric Disorders, Xiangya Hospital, Central South University, Changsha, Hunan 410008, China; 3 Department of Urology, The Second Xiangya Hospital of Central South University, Changsha, Hunan 410011, China; 4 Department of Urology, Hunan Cancer Hospital and The Affiliated Cancer Hospital of Xiangya School of Medicine, Central South University, Changsha, Hunan 410013, China

**Keywords:** benign prostatic hyperplasia (BPH), lncRNA DNM3OS, miR-29a/29b, COL3A1, TGF-β1

## Abstract

Transforming growth factor-β1 (TGFβ1)-induced differentiation into and the activation of myofibroblasts have been regarded as critical events in benign prostatic hyperplasia (BPH); however, the underlying mechanisms of BPH pathogenesis remain unclear. Microarray profiling, STRING analysis, Kyoto Encyclopedia of Genes and Genomes (KEGG) pathway annotation, and Gene Ontology (GO) enrichment analysis were performed to confirm the candidate genes and long non-coding RNA (lncRNAs) related to BPH. Collagen Type III (COL3A1) was significantly upregulated by TGFβ1 in prostate stromal cells (PrSCs) and might be involved in DNM3OS function in myofibroblasts upon TGFβ1 stimulation. Upon TGFβ1 stimulation, COL3A1 protein was decreased by DNM3OS silencing. miR-29a and miR-29b could directly bind to the DNM3OS and COL3A1 3' untranslated region (UTR)s to negatively regulate their expression, and by serving as a competing endogenous RNAs (ceRNA), DNM3OS competed with COL3A1 for miR-29a/29b binding, therefore counteracting miR-29a/29b-mediated COL3A1 suppression. The effect of DNM3OS silencing on ECM components and TGFβ1 downstream signaling was similar to that of the TGFβ1 inhibitor SB431542. miR-361 could target DNM3OS and TGFβ1; DNM3OS competed for miR-361 binding to counteract miR-361-mediated TGFβ1 suppression. In conclusion, we identified DNM3OS as a specifically-upregulated lncRNA upon TGFβ1 stimulation in PrSCs; by serving as a ceRNA for the miR-29a/29b cluster and miR-361, DNM3OS eliminated miRNA-mediated suppression of COL3A1 and TGFβ1, thereby promoting TGFβ1-induced PrSC transformation into myofibroblasts.

## INTRODUCTION

Benign prostatic hyperplasia (BPH), one of most commonly seen diseases of the urinary system, has an incidence of up to 60% in men aged between 40 and 60 years and higher than 90% in men aged 80 and over [1, 2]. During BPH, the most typical pathological changes include the formation of nodules that are primarily located in the transition zone (TZ) of the prostate [3], resulting in the enlargement of the periurethral prostate followed by urethra contraction [4]. In addition, epithelial overproliferation and the appearance of the stromal region can be observed [5].

According to previous studies, BPH is an extremely complex process that can be affected by multiple factors, resulting in poorly understood underlying mechanisms. In addition to the imbalance between androgen and estrogen, growth factors have also been reported to participate in the occurrence of BPH. Produced by stromal cells, transforming growth factor-β1 (TGFβ1) is a direct predisposing factor for prostate stromal hyperplasia, which is involved in multiple processes during the development of BPH, including inflammation. As critical factors associated with BPH development, acute and chronic inflammation both play a key role in the BPH pathogenic process as manifested by increased inflammatory cell infiltration and production and release of cytokines and chemokines [6–9]. Chronic inflammation is related to the initiation and/or development of tissue fibrosis, which is characterized by increased number and activity of myofibroblasts, collagen deposition and remodeling of extracellular matrix (ECM) [10]. During the differentiation of fibroblasts to myofibroblasts, several changes in gene expression have been regarded as a signature of myofibroblasts, such as increased expression of Tnc, Lox1, ELN, COL3A1, and Tnfrsf12a [11, 12]. Consistent with these observations, TGFβ1 is one of the critical cytokines that induce fibroblasts to transform into myofibroblasts and promote fibrosis, during which the expression of COL1A1, COL3A1, and α-SMA is increased [13, 14]. Accordingly, the fibrosis process in the liver and kidney can be affected by multiple factors including growth factors, which induce cell differentiation into myofibroblasts [15, 16]. As the main producers of ECM, myofibroblasts contribute directly to renal fibrosis [17–20] and liver fibrosis [16, 21, 22]. Thus, we speculate that the differentiation into and the activation of myofibroblasts play a critical role in BPH pathogenesis.

In addition to hormonal and inflammatory mechanisms, genetic mechanisms have also been shown to be common pathophysiological driving mechanisms for the development of BPH [23]. Several studies have aim to identify of genomic loci that confer risk for BPH; however, most of these studies have focused on protein coding regions which comprise less than 2% of the genome. During the last few decades, it has been demonstrated that at least 90% of the genome is actively transcribed into noncoding RNAs (ncRNAs) consisting of both microRNAs and long noncoding RNA (lncRNA), both of which play a fundamental role in both normal development and pathogenic processes [24]. LncRNAs can serve as competing endogenous RNAs (ceRNAs), which compete with miRNA for binding to target mRNAs, therefore regulating miRNA availability targeted mRNAs [25, 26]. Together, these molecules have been shown to participate in many aspects of cellular functions including stem cell pluripotency, cell-cycle regulation, chromatin remodeling, dosage compensation, genomic imprinting, cell differentiation, organogenesis and angiogenesis [27–29]. The deregulation of ncRNAs has been observed in several disorders, including BPH [30, 31]. Recent application of RNA sequencing (RNA-Seq) on urinary system-related disorders revealed a new list of dysregulated lncRNAs and miRNAs with a potential diagnostic and treatment value [32, 33].

In the present study, we first analyzed several online microarray profiles reporting lncRNAs specifically highly-expressed in prostate stroma, lncRNAs related to BPH, and lncRNAs that could be regulated by TGFβ1. Among the candidate lncRNAs, DNM3OS was examined for its expression in response to TGFβ1 in prostate stromal cells (PrSCs) and selected for further experiments. Next, we performed microarray profiling analyses on PrSCs with or without TGFβ1 treatment to identify differentially-expressed genes. The differentially-expressed genes were applied for Protein-Protein Interaction Networks (STRING) analysis, KEGG pathway annotation, and Gene Ontology enrichment analysis (GO analysis) to find the key factor(s) involved in myofibroblast differentiation during BPH, based on which COL3A1 was selected for further experiments. The effect of DNM3OS on COL3A1 expression was examined in PrSCs. Subsequently, miRNAs that might target DNM3OS and COL3A1 were predicted by online tools and miR-29a/b were selected, after which the predicted bindings of miR-29a/b to DNM3OS and COL3A1 were validated. Afterward, the dynamic effects of DNM3OS and miR-29a/b on COL3A1, ECM proteins, and the downstream factors of TGFβ1 were evaluated. Regarding another possible mechanism, we searched for miRNAs that might target both DNM3OS and TGFβ1 and miR-361 was identified; similarly, the predicted bindings and the dynamic effects of DNM3OS and miR-361 on TGFβ1, ECM proteins, and the downstream factors of TGFβ1 were evaluated. Finally, the expression and the correlations of these factors were examined and analyzed. In conclusion, we identified a lncRNA, namely, DNM3OS, which is specifically highly-expressed in prostate stroma and TGFβ1-stimulated PrSCs, and demonstrated two different miRNA/mRNA axes through which DNM3OS exerts its effects on ECM remodeling during BPH.

## RESULTS

### Selection of lncRNAs associated with benign prostatic hyperplasia (BPH) stroma and highly-expressed in prostate stromal tissues

**Figure 1:**
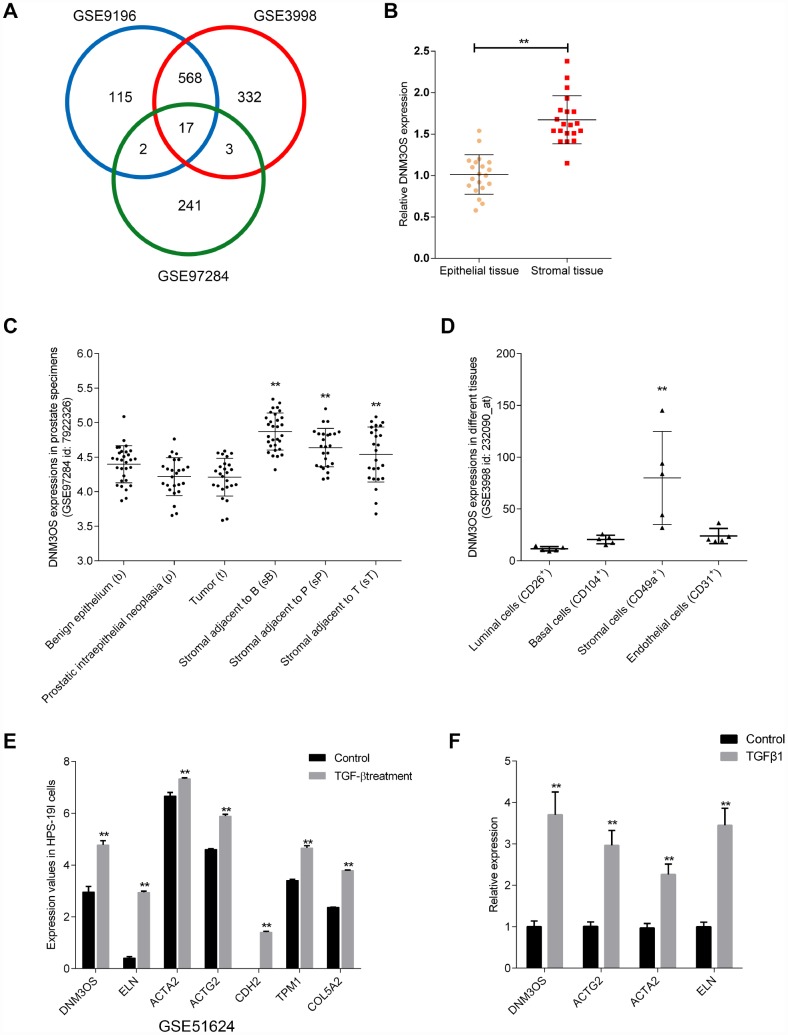
Selection of lncRNAs associated with benign prostatic hyperplasia (BPH) stroma and highly-expressed in prostate stromal tissues (**A**) Three microarray profiles reported differentially-expressed lncRNAs in prostate stromal tissues compared to prostatic epithelium (GSE9196, GSE3998, and GSE97284). The expression of lncRNA DNM3OS in (**B**) epithelial and stroma tissues according to GSE9196; (**C**) benign epithelium, prostatic intraepithelial neoplasia, tumors, stroma adjacent to benign epithelium, stroma adjacent to prostatic intraepithelial neoplasia, and stromal adjacent to tumor according to GSE97284; and (**D**) luminal cells, basal cells, stromal cells, and endothelial cells. (**E**) Differentially- expressed genes in the benign stromal cell line HPS-19I upon TGFβ treatment according to GSE51624. (**F**) Differentially- expressed genes in primary prostate stromal cells (PrSCs) upon TGFβ treatment. *P<0.05, **P<0.01.

To identify lncRNAs that are related to BPH, we downloaded the microarray profiles GSE9196, GSE3998, and GSE97284 from GEO to investigate specifically-upregulated lncRNAs in prostate stroma, compared to prostate epithelium (log_2_FC >= 0.56, *P* < 0.05). As shown in Figure 1A, a total of 17 lncRNAs were identified in all three microarray profiles to be upregulated in prostate stroma that included FGF7P3, FGF7P2, MEG8, RF00019, FGF7P5, FGF7P4, FGF7P1, DNM3OS, MIR99AHG, GBP1P1, CARMN, MEG3, FGF7P8, SNORD114-3, FGF7P6, CES1P1 and DIO3OS. The expression of these 17 lncRNAs in prostate stromal and epithelial tissue samples was examined. As shown in Figure 1B and Supplementary Figure 1A–1B, the expression of MEG8, FGF7P4, GBP1P1, FGF7P6, DIO3OS, and DNM3OS were significantly upregulated in prostate stromal tissues, and DNM3OS expression was the most upregulated.

Next, we compared DNM3OS expression in magnetic-activated cell sorting (MACS)-isolated prostate stromal cells (CD49a+) and prostate endothelial cells (CD31+) based on GSE9196 (Supplementary Figure 1C); in 30 cases of benign epithelium, prostatic intraepithelial neoplasia, tumors, and adjacent stromal tissues based on GSE97284 (Figure 1C); and in MACS-isolated luminal cells (CD26+), basal cells (CD104+), stromal cells (CD49a+), and endothelial cells (CD31+) from normal prostate tissues based on GSE3998 (Figure 1D) and found that DNM3OS expression was specifically upregulated in stromal tissues/cells. More importantly, according to GSE51624, the expression of DNM3OS in the prostate stromal cell line, HPS-19I, increased rapidly with TGFβ induction (Figure 1E). Concurrently, the expression of myofibroblast markers were also increased, including ACTG2, ACTA2, CDH2 (Figure 1E). After treatment with TGFβ1, the expression of DNM3OS in PrSCs was rapidly increased (Figure 1F). These results indicate that DNM3OS expression can be induced by TGFβ and may be involved in stromal cell proliferation and BPH. Thus, lncRNA DNM3OS was selected for further experiments.

### Microarray profiling analysis differentially-expressed genes in PrSCs induced by TGFβ1

**Figure 2:**
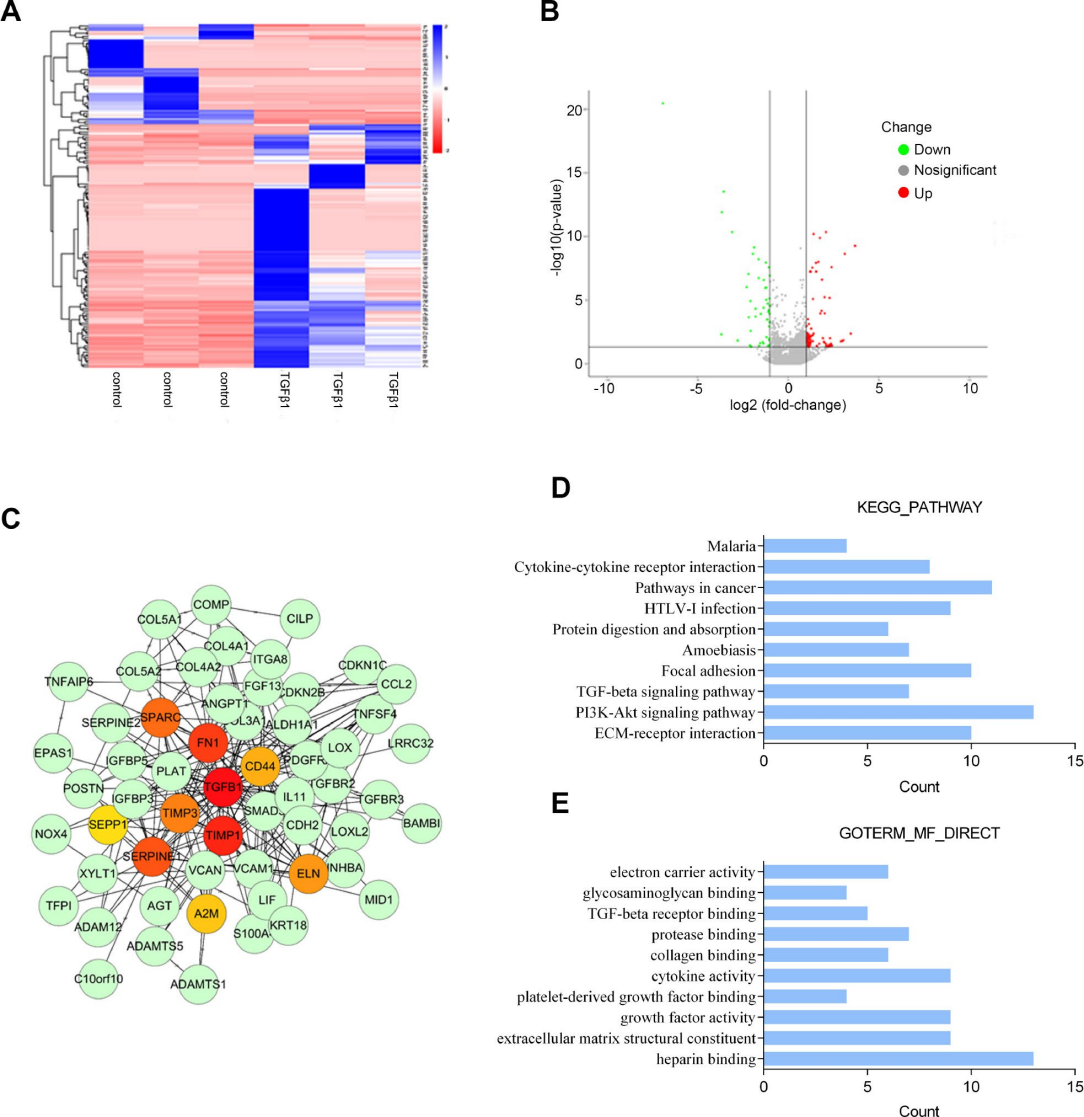
Microarray profile analysis of differentially-expressed genes in PrSCs induced by TGFβ1 analyzed by (**A**) Hierarchical clustering of gene expression in PrSCs with or without TGFβ1 treatment. (**B**) Volcano plot showing the differentially- expressed genes. (**C**) Network diagram of the interaction between upregulated and downregulated genes constructed by STRING analysis and visualized by Cytoscape. (**D**) KEGG pathway annotation of the differentially-expressed genes. (**E**) GO enrichment analyses of the differentially-expressed genes.

To identify differentially-expressed genes in PrSCs in response to TGFβ1 stimulation, we performed microarray profiling analyses. Hierarchical clustering analyses represented the expression of differentially-expressed genes in PrSCs with or without TGFβ1 treatment (Figure 2A). The distribution of gene expression differences is demonstrated as a volcano plot in Figure 2B. The volcano plot showed obvious differences in the distribution of differentially-expressed genes between two samples. The abscissa indicates the fold-change (shown as log_2_ fold-change). The ordinate indicates the p-value in the *t*-test (shown as -log_10_ p-value). The more significant the difference was the farther the numerical point is from the origin point. Thus, the most significantly differentially-expressed genes were distributed in the upper left and upper right corners. The most prominent gene for up-regulation is COL3A1. COL3A1 is a key marker of myofibroblasts and an important factor involved in TGFβ-mediated remodeling of the ECM; its expression was increased by at least 2 times (Log_2_FC ≥ 1, *P* < 0.05). Next, we used the STRING database to establish a protein-protein interaction (PPI) network of 108 key upregulated and downregulated genes. Network visualization was conducted by Cytoscape and the key nodes were analyzed using the Hub gene plug-in in Cytoscape. We found that 10 genes, that included TGFB1, CD44, FN1, SPARC, TIMP1, TIMP3, SEPPINE1, ELN, A2M and CD44, were at the core position of the PPI network comprised of the 108 genes (Figure 2C). KEGG signaling pathway annotation indicated that these genes were most enriched in the PI3K/AKT, cancer-related, ECM-receptor interaction, and focal adhesion signaling pathways (Figure 2D). GO enrichment analyses indicated that these genes were the most enriched in heparin binding, cytokine activity, growth factor activity, and extracellular matrix structural constituent (Figure 2E). Based on these data, COL3A1 was selected for further experiments due to its close association with TGFβ-mediated differentiation into and activation of myofibroblasts.

### DNM3OS silencing decreases the protein level of COL3A1 upon TGFβ1 stimulation

**Figure 3:**
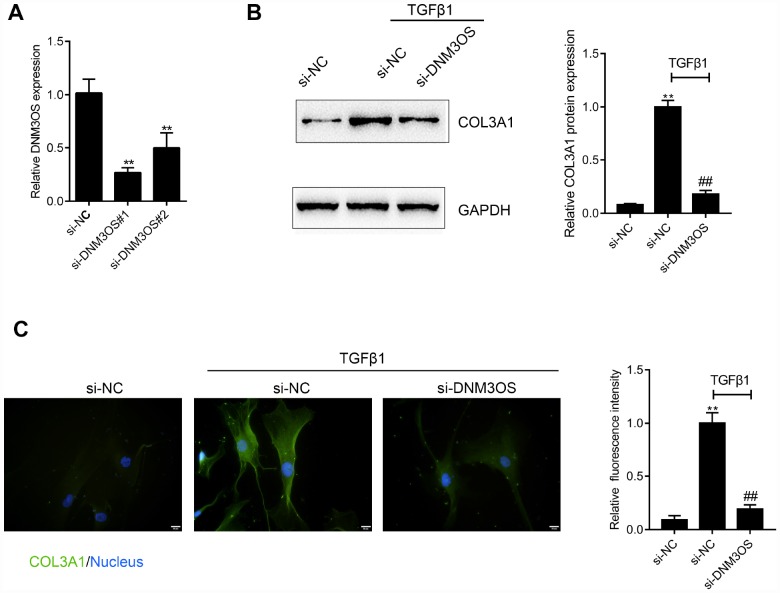
DNM3OS silencing decreases the protein level of COL3A1 (**A**) DNM3OS silencing conducted in PrSCs by transfection of si-DNM3OS#1 or si-DNM3OS#2 and confirmed by real-time PCR. PrSCs were transfected with si-DNM3OS in the presence or absence of TGFβ1 and examined for (**B**) the protein level of COL3A1 by Immunoblotting and (**C**) the protein content and distribution of COL3A1 by immunofluorescence (IF) staining (scale bar: 20 μM). *P<0.05, **P<0.01 compared to the si-NC group, ## *P*<0.01 compared to si-NC+TGFβ1 group.

We showed that DNM3OS and COL3A1 expression could be induced by TGFβ. Next, we investigated the effect of DNM3OS on COL3A1 upon TGFβ1 stimulation. Since DNM3OS is specifically upregulated in prostate stroma tissues and cells, we conducted DNM3OS silencing in PrSCs by transfection of si-DNM3OS#1 or si-DNM3OS#2, and based on real-time PCR data, si-DNM3OS#1 was selected for its better transfection efficiency (Figure 3A). Next, si-NC (negative control, scramble RNA sequence) or si-DNM3OS-transfected PrSCs were examined for COL3A1 protein levels and distribution with or without TGFβ1 stimulation. As shown in Figure 3B and 3C, TGFβ1 stimulation significantly increased the protein level of COL3A1, compared to that in the nontreated group, while the TGFβ1-induced increase in COL3A1 protein was decreased by DNM3OS silencing. These data indicate that DNM3OS can affect the expression of COL3A1 upon TGFβ1 stimulation.

### miR-29a and miR-29b directly bind DNM3OS and COL3A1 3'UTR to negatively regulate their expression

To identify the miRNAs that might be related to DNM3OS and COL3A1 expression in PrSCs, we used the online prediction tool Target Scan to predict miRNAs that may target COL3A1; a total of 313 candidate miRNAs were predicted, 24 of which had conserved binding sites. Among the 24 miRNAs, two were predicted by LncTar to bind lncRNA DNM3OS, namely, miR-29a-3p and miR-29b-3p. Next, we investigated whether DNM3OS could serve as a ceRNA for miR-29a/29b to counteract miR-29a/29b-mediated COL3A1 suppression.

**Figure 4:**
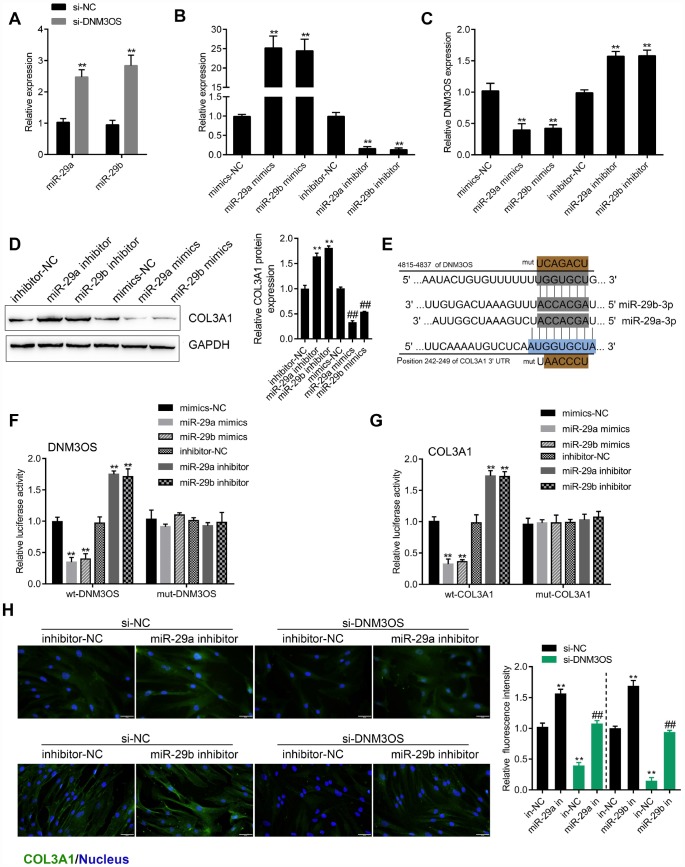
miR-29a and miR-29b directly bind the DNM3OS and COL3A1 3'UTR to negatively regulate their expression (**A**) PrSCs were transfected with si-DNM3OS and examined for the expression of miR-29a/29b by real-time PCR. (**B**) miR-29a/29b overexpression or inhibition conducted in PrSCs by transfection of miR-29a/29b mimics or inhibitor and confirmed by real-time PCR. (**C**) PrSCs were transfected with miR-29a/29b mimics or inhibitor and examined for the expression of DNM3OS by real-time PCR. (**D**) PrSCs were transfected with miR-29a/29b mimics or inhibitor and examined for the protein levels of COL3A1. (**E**) A schematic diagram showing the predicted binding sites between miR-29a/29b and DNM3OS or COL3A1. Wild- and mutant-type DNM3OS or COL3A1 3'UTR luciferase reporter vectors were constructed. Mutant-type vectors contained a 7-bp mutation in the predicted miR-29a/29b binding site. (**F**–**G**) 293T cells were cotransfected with these vectors and miR-29a/29b mimics or inhibitor and examined for luciferase activity. (**H**) PrSCs were cotransfected with si-DNM3OS and miR-29a/29b inhibitor and examined for the protein content and distribution of COL3A1 by IF staining (scale bar: 50 μM). **P<0.01.

The expression of miR-29a and miR-29b was significantly upregulated in DNM3OS-silenced PrSCs (Figure 4A). To examine the effects of miR-29a/29b on DNM3OS expression, we conducted miR-29a/29b overexpression or inhibition by transfection of PrSCs with miR-29a/29b mimics or inhibitor, as confirmed by real-time PCR (Figure 4B). In PrSCs, DNM3OS expression was negatively regulated by miR-29a and miR-29b (Figure 4C). Consistent with online tool prediction, miR-29a and miR-29b negatively regulated the protein levels of COL3A1 (Figure 4D).

To validate the predicted binding interactions, we performed luciferase reporter assays. Wild- and mutant-type DNM3OS and COL3A1 3'UTR luciferase reporter vectors were constructed as described in the M&M section (Figure 4E). These vectors were cotransfected in 293T cells along with miR-29a/29b mimics or inhibitor and then the luciferase activity was examined. As shown in Figure 4F–4G, the luciferase activity of wt-DNM3OS/COL3A1 3'UTR was significantly suppressed by miR-29a/29b overexpression but enhanced by miR-29a/29b inhibition, while the changes in the luciferase activity were abolished after mutating the predicted binding site. These data indicate that miR-29a/29b can directly bind the DNM3OS and COL3A1 3'UTR to negatively regulate their expression.

After confirming the binding of miR-29a/29b to the DNM3OS and COL3A1 3'UTR, next, we examined whether DNM3OS competes with COL3A1, therefore serving as a ceRNA for miR-29a/29b and counteracting the miR-29a/29b-mediated COL3A1 suppression. PrSCs were cotransfected with si-DNM3OS and miR-29a/29b inhibitor and examined for the protein content of COL3A1 by IF staining. As shown in Figure 4H, DNM3OS silencing significantly decreased, while miR-29a/29b inhibition increased the protein content of COL3A1; the effects of DNM3OS silencing on COL3A1 protein could be partially reversed by miR-29a/29b inhibition.

### Effects of DNM3OS silence and SB431542 on ECM components and TGFβ1 downstream signaling are similar

**Figure 5:**
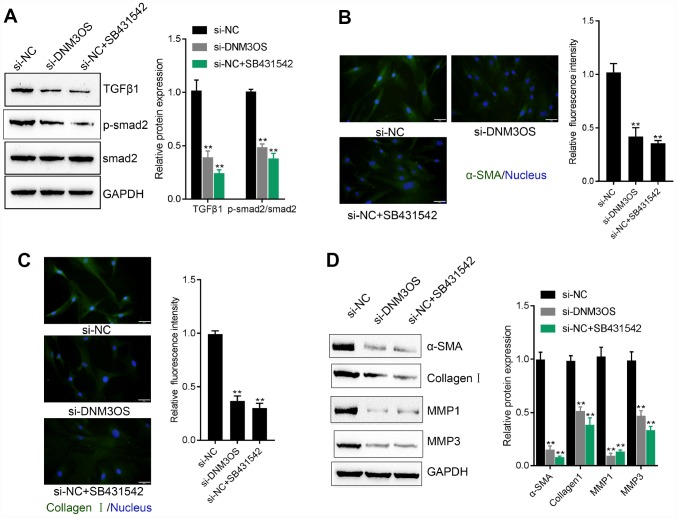
Similar effects of DNM3OS silencing and SB431542 on ECM components and TGFβ1 downstream signaling PrSCs were transfected with si-DNM3OS or treated with the TGFβ1 inhibitor SB431542 and examined for (**A**) the protein levels of TGFβ1, p-Smad2, and Smad2 by immunoblotting; (**B**–**C**) the protein content and distribution of α-SMA and Collagen I by IF staining (scale bar: 50 μM) and (**D**) the protein levels of α-SMA, Collagen I, MMP1, and MMP3 by immunoblotting. **P<0.01.

As we have mentioned, COL3A1 is an important factor involved in TGFβ-mediated myofibroblast functions. Next, we investigated whether DNM3OS could modulate TGFβ1 downstream signaling and TGFβ1-mediated ECM component changes. PrSCs were transfected with si-DNM3OS or treated with SB431542, an inhibitor of TGFβ1, and examined for the protein levels of TGFβ1, p-Smad2, Smad2, α-SMA, Collagen I, MMP1, and MMP3. As shown in Figure 5A–5D, DNM3OS silencing significantly decreased the protein levels of these factors, as revealed by immunoblotting and IF staining, which was similar to the effect of SB431542. These data indicate that, in addition to its effect on the miR-29a/29b-COL3A1 axis, DNM3OS might also modulate TGFβ1 to exert its effects on PrSCs.

### miR-361 directly binds DNM3OS and TGFβ1 to inhibit their expression

**Figure 6:**
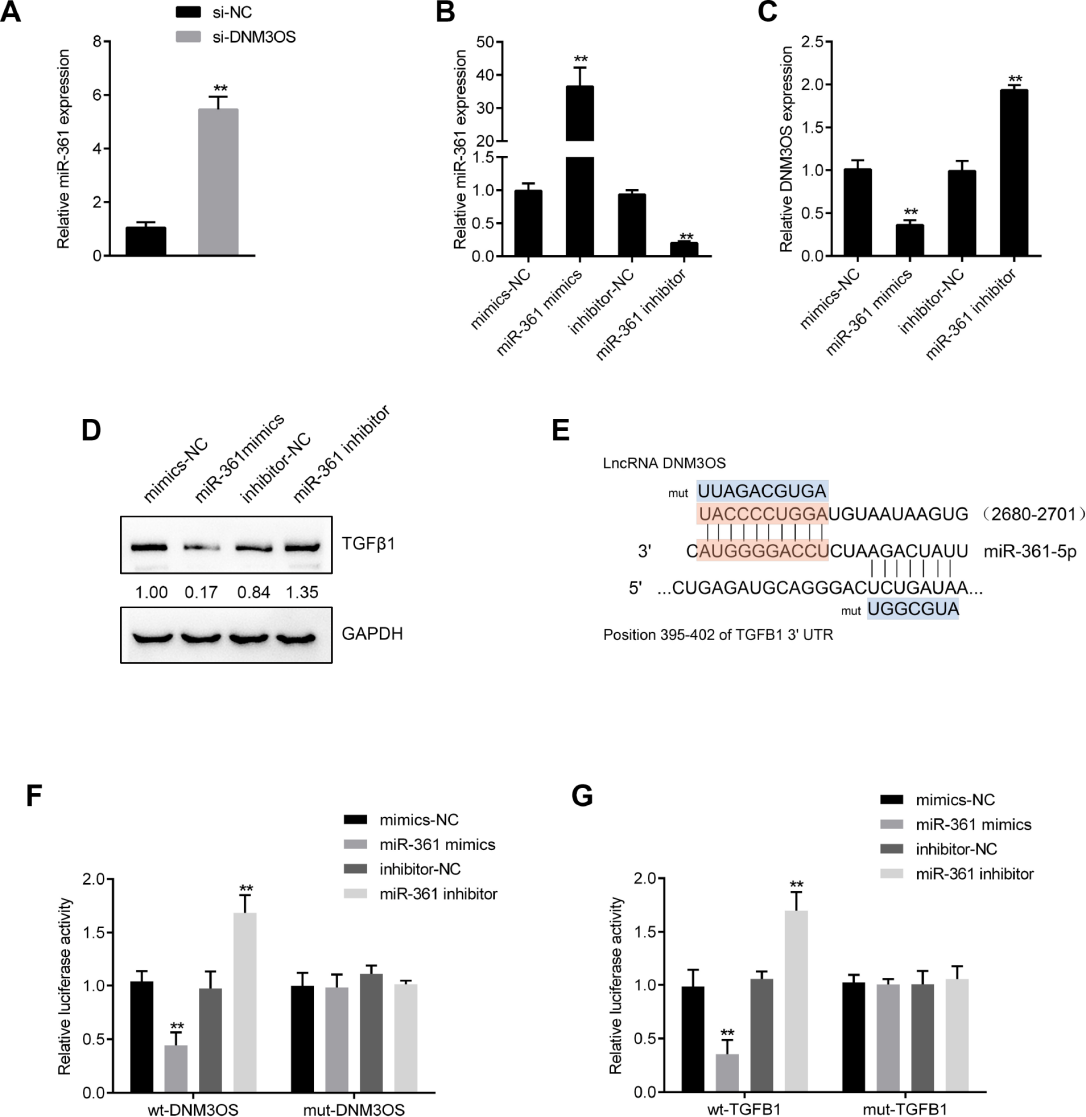
DNM3OS competes for miR-361 binding to counteract miR-361-mediated TGFβ1 suppression (**A**) PrSCs were transfected with si-DNM3OS and examined for the expression of miR-361 by real-time PCR. (**B**) miR-361 overexpression or inhibition conducted in PrSCs by transfection of miR-361 mimics or inhibitor, as confirmed by real-time PCR. (**C**) PrSCs were transfected with miR-361 mimics or inhibitor and examined for the expression of DNM3OS by real-time PCR. (**D**) PrSCs were transfected with miR-361 mimics or inhibitor and examined for the protein levels of TGFβ1. (**E**) A schematic diagram showing the predicted binding sites between miR-361 and DNM3OS or TGFβ1. Wild- and mutant-type DNM3OS or TGFβ1 3'UTR luciferase reporter vectors were constructed. Mutant-type vectors contained a 7- or 10- bp mutation in the predicted miR-361 binding site. (**F**–**G**) 293T cells were cotransfected with the vectors and miR-361 mimics or inhibitors and examined for luciferase activity. **P<0.01.

To investigate another possible mechanism by which DNM3OS exerts its effects, we used online tools to predict miRNAs that might target DNM3OS and TGFβ1. A total of 6 miRNAs were predicted to have conserved binding sites and only miR-361 was predicted by both TargetScan and LncTar to target DNM3OS and TGFβ1. Similar to the results for miR-29a/29b, DNM3OS silencing significantly upregulated miR-361 expression (Figure 6A). To examine the effect of miR-361, we conducted miR-361 overexpression and inhibition in PrSCs by transfection of miR-361 mimics or inhibitor, as confirmed by real-time PCR (Figure 6B). In PrSCs, miR-361 negatively regulated DNM3OS expression (Figure 6C) and TGFβ1 protein levels (Figure 6D).

To validate the predicted binding interactions, we performed luciferase reporter assays by constructing wild- and mutant-type DNM3OS and TGFβ1 3'UTR luciferase reporter vectors as described in the M&M section (Figure 6E). After cotransfection with miR-361 mimics or inhibitor, luciferase activity was determined. miR-361 overexpression significantly suppressed, while miR-361 inhibition enhanced the luciferase activity of the wild-type vectors, and after mutating the predicted binding site, the changes in luciferase activity were abolished (Figure 6F–6G). These data indicate that miR-361 could directly bind the DNM3OS and TGFβ1 3'UTR to negatively regulate their expression.

### The dynamic effects of DNM3OS and miR-361 on TGFβ1 and downstream signaling

**Figure 7:**
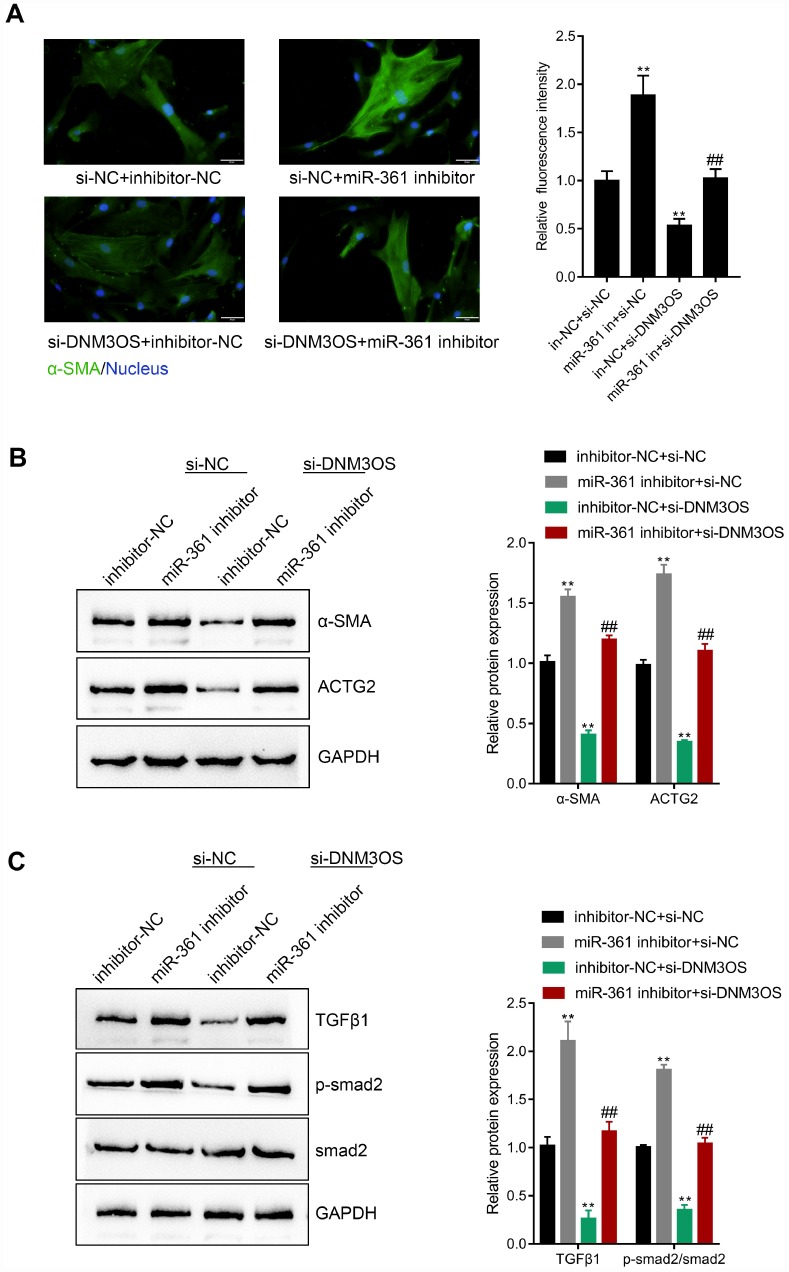
The dynamic effects of DNM3OS and miR-361 on TGFβ1 and downstream signaling PrSCs were cotransfected with si-DNM3OS and miR-361 inhibitor and examined for (**A**) the protein content and distribution of α-SMA by IF staining (scale bar: 50 μM); (**B**) the protein levels of α-SMA and ACTG2 by immunoblotting and (**C**) the protein levels of TGFβ1, p-Smad2, and Smad2 by immunoblotting.

After confirming the predicted binding of miR-361 to DNM3OS and TGFβ1, we validated whether DNM3OS exerts its effects on TGFβ1, ECM components, and TGFβ1 downstream signaling via miR-361. PrSCs were cotransfected with si-DNM3OS and miR-361 inhibitor and examined for the protein levels of α-SMA, ACTG2, TGFβ1, p-Smad2, and Smad2. As shown in Figure 7A–7C, miR-361 inhibitor increased, while DNM3OS silencing decreased the protein levels of these factors and the effects of DNM3OS silencing could be reversed by miR-361 inhibition. These data indicate that DNM3OS also serves as a ceRNA for miR-361 to counteract miR-361-mediated TGFβ1 suppression.

### The expression and correlation of DNM3OS, miR-29a/29b/361, COL3A1, and TGFβ1 in tissue samples

**Figure 8:**
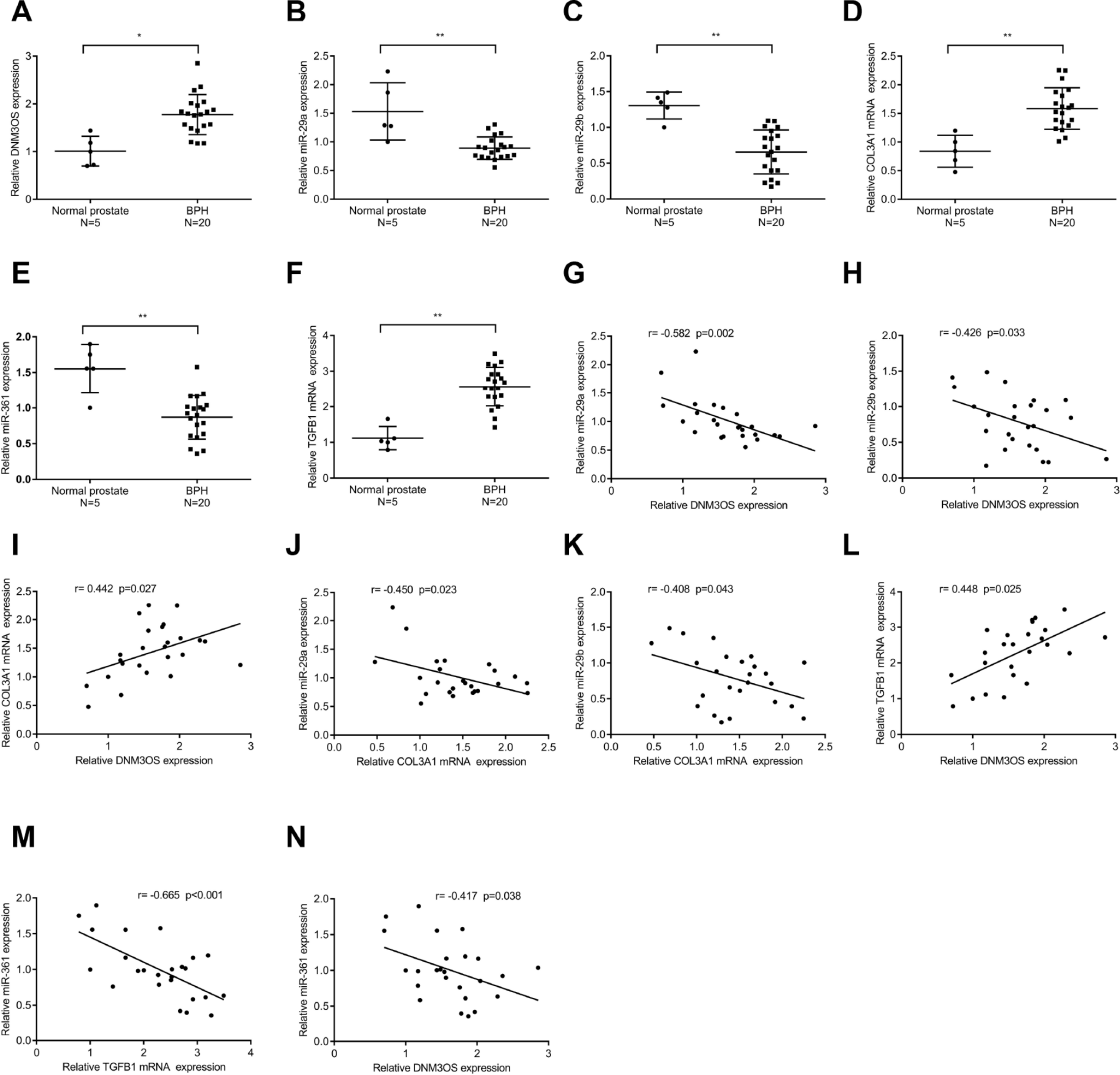
The expression and correlation of DNM3OS, miR-29a/29b/361, COL3A1, and TGFβ1 in tissue samples (**A**–**F**) The expression of DNM3OS, miR-29a/29b/361, COL3A1, and TGFβ1 in normal prostate and BPH tissue samples (n = 20) determined by real-time PCR. (**G**–**N**) The correlation of DNM3OS, miR-29a/29b/361, COL3A1, and TGFβ1 in tissue samples analyzed by Pearson’s correlation analyses.

To further confirm the above findings, we examined the expression of DNM3OS, miR-29a/29b/361, COL3A1, and TGFβ1 in normal prostate stromal and BPH tissue samples. As shown in Figure 8A–8F, the expression of DNM3OS, COL3A1, and TGFβ1 was significantly upregulated, while miR-29a, miR-29b, and miR-361 expression was significantly downregulated in BPH tissues, compared to normal prostate tissues. Moreover, DNM3OS was negatively correlated with miR-29a, miR-29b, and miR-361; COL3A1 was negatively correlated with miR-29a and miR-29b; TGFβ1 was negatively correlated with miR-361; and DNM3OS was positively correlated with COL3A1 and TGFβ1 (Figure 8G–8N). These data indicate that DNM3OS could promote TGFβ1-mediated myofibroblast activation in PrSCs by counteracting the miR-29a/29b-mediated COL3A1 suppression and miR-361-mediated TGFβ1 suppression.

## DISCUSSION

In the present study, we analyzed online microarray profiles to identify lncRNAs that might be involved in TGFβ1-mediated myofibroblast differentiation and activation and DNM3OS was selected for further analysis. Based on our microarray profiling analysis of differentially-expressed genes in PrSCs treated with or without TGFβ1, COL3A1 was significantly upregulated by TGFβ1 and could be involved in DNM3OS function in myofibroblasts upon TGFβ1 stimulation. Upon TGFβ1 stimulation, COL3A1 protein was decreased by DNM3OS silencing. Using online tools prediction and experimental analyses, two miRNAs, miR-29a and miR-29b were identified to directly bind to the DNM3OS and COL3A1 3'UTRs to negatively regulate their expression. By serving as a ceRNA, DNM3OS competed with COL3A1 for miR-29a/29b binding, thereby counteracting the miR-29a/29b-mediated COL3A1 suppression. More importantly, the effect of DNM3OS silencing on ECM components and TGFβ1 downstream signaling was similar to that of the TGFβ1 inhibitor SB431542. Regarding the molecular mechanism, miR-361 could target DNM3OS and TGFβ1 according to the online tool prediction and experimental results, while DNM3OS competed for miR-361 binding to counteract the miR-361-mediated TGFβ1 suppression. In summary, DNM3OS could promote TGFβ1-mediated myofibroblast activation in PrSCs by counteracting miR-29a/29b-mediated COL3A1 suppression and miR-361-mediated TGFβ1 suppression.

PrSCs differentiation into myofibroblasts and the activation of myofibroblasts play a crucial role in BPH pathogenesis [34, 35]; molecular interventions inducing dysfunction in myofibroblasts could offer a novel therapeutic strategy. TGFβ1 is considered to be a key inducer of pathogenic stromal reorganization, and could induce fibroblast-to-myofibroblast trans-differentiation in PrSCs [34, 36, 37], which is regarded as a basic property of BPH [38–40]. In view of the potent roles of ncRNAs in several disorders, including in BPH [30, 31], in the present study we monitored the different expressed lncRNAs in PrSCs upon TGFβ1 treatment, and found that the expression of DNM3OS, which was specifically upregulated in stromal tissues, was significantly promoted by TGFβ1 stimulation. LncRNA DNM3OS was first identified as a 6-kb antisense transcript contained within an intron of the mouse Dnm3 gene by Loebel et al. [55] in 2005. Although the mouse DNM3OS gene (GenBank: AB159607) has a potential open reading frame, a comparison of the sequences found in several higher vertebrates revealed that the predicted ATG start site of the mouse DNM3OS gene was not conserved in the human or chicken sequences despite the overall sequence conservation especially in the region of the 5′ end of the transcript. Therefore, DNM3OS was considered to be a ncRNA [56]. Interestingly, DNM3OS has been reported in several fibrosis diseases. During peritendinous fibrosis, TGFβ1 treatment significantly increased DNM3OS expression and the viability of primary tenocytes; after DNM3OS silencing in tenocytes, the TGFβ1-induced upregulation of fibrogenesis-related factors, including collagen I, collagen III, α-SMA, and FN 1 was significantly decreased [41]. Savary et al. [42] also demonstrated that DNM3OS is involved in the TGF-β-induced activation of lung myofibroblasts serving as a fibroblast-specific effector, which could induce miR-199a-5p, miR-199a-3p, and miR-214-3p expression. Thus, it is reasonable to speculate that DNM3OS plays a critical role in the transformation of PrSCs to myofibroblasts upon TGFβ1 stimulation, therefore affecting BPH.

Along with TGFβ1-induced DNM3OS upregulation, a significant increase in COL3A1 expression upon TGFβ1 stimulation also drew our attention. Reportedly, the increase in the transcriptional levels of collagen I and collagen III in the bladder are related to BPH pathogenesis [43]. During BPH, hypertrophy of smooth muscle cells and enhanced ECM deposition have been regarded as mechanisms of bladder thickness increase and compliance loss. An imbalance in collagen production and degradation can lead to ECM deposition, a process that appears to be mediated by different events, including increases in collagen I and collagen III [44, 45]. In the present study, upregulation of DNM3OS and COL3A1 in response to TGFβ1 stimulation was observed in PrSCs. Moreover, the TGFβ1-induced increase in COL3A1 protein could be significantly decreased by DNM3OS silencing, suggesting that DNM3OS might exert its effects in BPH in a COL3A1-related manner.

Commonly, lncRNAs exert their functions by serving as ceRNAs to compete with mRNAs for miRNA binding, thus influencing the available level of miRNAs [25]. Since DNM3OS positively regulated COL3A1 protein levels upon TGFβ1 stimulation, we hypothesized that miRNAs might be involved in this process. As revealed by online tools and experimental analyses, two miRNAs, namely, miR-29a and miR-29b, could directly target DNM3OS and the 3'UTR of COL3A1. Reportedly, the miR-29a/29b cluster plays a fibrosis-suppressive role. By targeting Notch2, miR-29a/29b suppresses high glucose-induced endothelial-mesenchymal transition (EMT) in human retinal microvascular endothelial cells [46]. In Sprague-Dawley rats, miR-29a inhibits cardiac fibrosis by downregulating the expression of DNMT3A [47]. In addition, miR-29a has been identified as a key regulon of BRD4 regulation and liver fibrosis decrease in mice by inhibiting hepatic stellate cell activation [48]. In the present study, miR-29a or miR-29b inhibition both significantly reversed the effects of DNM3OS silencing on COL3A1 expression. TGFβ1 significantly induced the increase in COL3A1, while DNM3OS silencing reversed the induction effects of TGFβ1 on COL3A1. In addition, knocking down DNM3OS alone could decrease COL3A1 expression. These data indicate that DNM3OS could regulate COL3A1 expression with or without TGFβ1 treatment. The binding between DNM3OS and miR-29a/miR-29b, as well as between miR-29a/miR-29b and COL3A1 is ubiquitous; thus, the mechanism by which DNM3OS silencing can decrease COL3A1 expression with or without TGFβ1 is that DNM3OS serves as a ceRNA for miR-29a/miR-29b to counteract miR-29a/miR-29b-mediated COL3A1 suppression.

As we have mentioned, TGFβ1 is one of the critical cytokines that induce fibroblasts to transform into myofibroblasts and promote fibrosis, during which the expression of COL1A1, COL3A1, and α-SMA is increased [13]. Interestingly, we observed that the effects of DNM3OS silencing and SB431542 on ECM components and TGFβ1 signaling were similar, that is, DNM3OS silencing significantly inhibited the phosphorylation of Smad2 and decreased the protein levels of TGFβ1, Collagen I, α-SMA, MMP-1, and MMP3. These data indicate that DNM3OS might also positively regulate TGFβ1 in a miRNA-related manner. Similarly, online tools and experimental analyses revealed that miR-361 could target DNM3OS and TGFβ1 to inhibit their expression. Regarding the specific effects, miR-361 inhibition could reverse the effects of DNM3OS silencing on the protein levels of α-SMA, ACTG2 and TGFβ1, as well as the phosphorylation of Smad2, indicating that DNM3OS could serve as a ceRNA for miR-361 to counteract miR-361-mediated suppression of TGFβ1, thereby enhancing the effects of TGFβ1 stimulation on PrSCs.

## CONCLUSIONS

In conclusion, we identified DNM3OS as a specifically-upregulated lncRNA upon TGFβ1 stimulation in PrSCs; via serving as a ceRNA for miR-29a/29b cluster and miR-361, DNM3OS abolished the miRNA-mediated suppression of COL3A1 and TGFβ1, thereby promoting TGFβ1-induced PrSC transformation into myofibroblasts (Graphical abstract).

**Figure 9:**
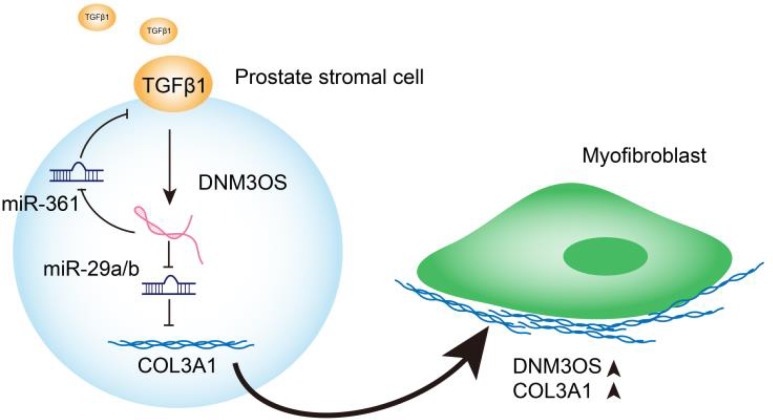
**Graphical abstract:** A schematic diagram of the proposed mechanisms of LncRNA DNM3OS-mediated TGFβ1-induced PrSC transformation into myofibroblasts.

## MATERIALS AND METHODS

### Clinical samples

Male patients (n=20, 67.1±4.8 years) that had been diagnosed with BPH and had undergone transurethral resection of the prostate (TURP) at Xiangya Hospital were recruited with approval by the Ethics Committee of Xiangya Hospital. As normal prostate controls, normal prostate tissues were obtained from 5 bladder tumor patients (40.1±2.4 years) undergoing radical cystoprostatectomy. Written informed consent for participation in the study was obtained from all patients involved. The collected prostate samples were stored in liquid nitrogen or paraffin until further experiments.

### Cell lines, cell culture, and cell transfection

PrSCs were primary cultured from BPH tissues according to procedures described previously [49] and cultured in RPMI-1640 medium (Gibco, Waltham, MA, USA) supplemented with 10% FBS (Invitrogen, Waltham, MA, USA) and 1% penicillin-streptomycin. Cells were cultured at 37°C in 5% CO_2_. Cells from passages 3~4 were used [50].

Silencing of lncRNA DNM3OS was achieved by transfection of specific small interfering RNA si-DNM3OS#1 or si-DNM3OS#2 (GenePharma, Shanghai, China). Scramble sequence siRNA was used as negative control (si-NC). Expression of miR-29a/miR-29b/miR-361 was achieved by transfection of miR-29a/miR-29b/miR-361 mimics or miR-29a/miR-29b/miR-361 inhibitor (GenePharma) using Lipofectamine 3000 (Invitrogen).

### Microarray profiling of PrSCs with or without TGFβ1 treatment

Clariom™ D microarray analysis (Thermo Fisher Scientific, Waltham, MA, USA) was performed on prostate stromal cells with or without TGFβ1 treatment. To select the differentially expressed genes, we used threshold values of |Log_2_FC| ≥ 1 and a Benjamini-Hochberg corrected *P* value of 0.05. The data were processed and analyzed following the methods described previously [51]. Finally, these differentially-expressed genes were applied for Protein-Protein Interaction Networks (STRING) analysis, KEGG pathway annotation (https://www.genome.jp/kegg/), and Gene Ontology enrichment analysis (GO analysis), and the visualization was achieved by using Cytoscape [52].

### RNA isolation and real-time PCR

Total RNA was extracted using TRIzol reagent (Invitrogen) and cDNA was synthesized by using a high-capacity cDNA reverse transcriptase kit (Thermo Fisher Scientific, Waltham, MA, USA) as previously described [53]. Relative RNA expression was detected using SYBR Green quantitative PCR reagent (Beijing TransGen Biotech, Beijing, China). Gene expression was analyzed using the 2^−ΔΔCt^ method [54].

### Immunoblotting

Using a Cell Mitochondria Isolation Kit (Beyotime, Shanghai, China), we extracted proteins from target cells. The samples were incubated with the following primary antibodies: anti-TGFβ1 (ab21610, Abcam, Cambridge, MA, USA), anti-TGFβ1 (ab64715, Abcam), anti-α-SMA (ab5694, Abcam), anti-p-SMAD2 (ab53100, Abcam), anti-SMAD2 (ab40855, Abcam), anti-MMP1 (ab52631, Abcam), anti-MMP3 (ab52915, Abcam), anti-ACTG2 (SAB1411364, Sigma-Aldrich, St. Louis, MO, USA), and anti-GAPDH (ab8245, Abcam) overnight at 4°C; afterward, the samples were incubated with goat anti-rabbit IgG polyclonal antibody (Abcam) or goat anti-mouse IgG polyclonal antibody (Abcam) for 1 h at room temperature. Visualization was conducted using an enhanced chemiluminescence (ECL) detection system (Thermo Fisher Scientific) using GAPDH levels as an endogenous control.

### Immunofluorescence (IF) staining

Cells were incubated with anti-TGFβ1 (ab21610, Abcam), anti-α-SMA (ab5694, Abcam), or anti-Collagen I (ab34710, Abcam) followed by a 60-min incubation with anti-IgG-FITC (Abcam) at room temperature. DAPI was then used for nuclear staining. Cells were then observed under a fluorescence microscope (Olympus, Japan) and representative images are shown.

### LncRNA-miRNA-mRNA correlations verified by luciferase reporter assay

To verify the predicted binding of lncRNA DNM3OS, miR-29a/29b/361, COL3A1, and TGFβ1, we performed a luciferase reporter assay by constructing wild-type and mutant-type DNM3OS or TGFβ1 3'UTR and COL3A1 3’UTR reporter vectors. For DNM3OS- miR-29a/29b-COL3A1, wild-type vectors contain wild DNM3OS fragment or COL3A1 3'UTR possessing the predicted miR-29a/29b binding site; mutant-type vectors contain mutated DNM3OS fragment or COL3A1 3'UTR possessing several bps mutations in the predicted miR-29a/29b binding site. These vectors were cotransfected with miR-29a/29b mimics or inhibitor. For DNM3OS-miR-361 -TGFβ1, wild-type vectors contain wild DNM3OS fragment or TGFβ1 3'UTR possessing the predicted miR-361 binding site; mutant-type vectors contained mutated DNM3OS fragment or TGFβ1 3'UTR possessing several bps mutations in the predicted miR-361 binding site. These vectors were cotransfected with miR-361 mimics or inhibitors. Then the changes in the luciferase activity were monitored using the Dual-Luciferase Reporter Assay System (Promega, Madison, MI, USA) 48 h after transfection. Renilla luciferase activity was normalized to firefly luciferase activity for each transfected well.

### Statistical analysis

Data from at least three independent experiments were processed using GraphPad software (San Diego, CA, USA) and then expressed as the means ± standard deviation (SD). Statistical methods used include one-way analysis of variance (ANOVA) followed by Tukey's multiple comparison test or independent sample *t*-test. A *P* value < 0.05 was considered as statistically significant.

### Ethical approval

All procedures performed in studies involving human participants were in accordance with the ethical standards of Xiangya Hospital and with the 1964 Helsinki declaration. Informed consent to participate in the study has been obtained from participants.

## Supplementary Material

Supplementary Figure 1
