# Supplementary material for: Long noncoding RNA DNM3OS promotes prostate stromal cells transformation via the miR-29a/29b/COL3A1 and miR-361/TGFβ1 axes
Source: Aging (Albany NY). 2019 Nov 6;11(21):9442–60. doi: 10.18632/aging.102395 (PMC6874426; doi:10.18632/aging.102395)
Supplement: Supplementary Figure 1 [file aging-11-102395-s001.pdf]

## SUPPLEMENTARY FIGURE

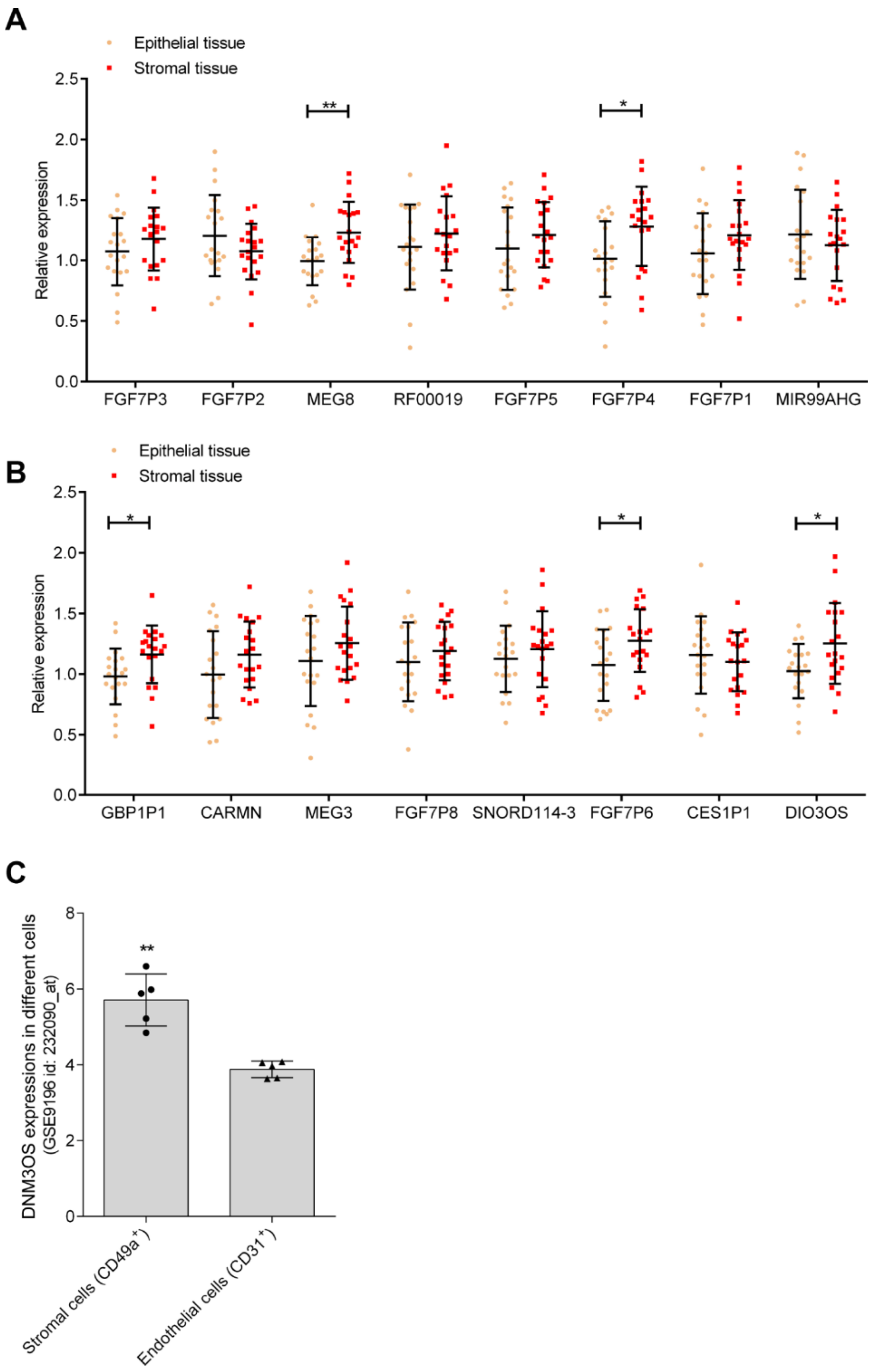


**Supplementary Figure 1. Expression of candidate lncRNAs in tissue samples or cells based on online data.**
